# Influence of Two Garlic-Derived Compounds, Propyl Propane Thiosulfonate (PTS) and Propyl Propane Thiosulfinate (PTSO), on Growth and Mycotoxin Production by *Fusarium* Species *In Vitro* and in Stored Cereals

**DOI:** 10.3390/toxins11090495

**Published:** 2019-08-27

**Authors:** Kalliopi Mylona, Esther Garcia-Cela, Michael Sulyok, Angel Medina, Naresh Magan

**Affiliations:** 1 Applied Mycology Group, Environment and AgriFood Theme, Cranfield University, Cranfield MK43 0AL, UK; 2 Institute of Bioanalytics and Agro-Metabolomics, Department of Agrobiotechnology (IFA-Tulln), University of Natural Resources and Life Sciences, Vienna, Konrad Lorenzstr. 20, A-3430 Tulln, Austria

**Keywords:** *Fusarium*, mycotoxins, garlic-derived extracts, green chemistry, fungi, EU limits, abiotic factors, storage, wheat, maize, oats

## Abstract

Two garlic-derived compounds, Propyl Propane Thiosulfonate (PTS) and Propyl Propane Thiosulfinate (PTSO), were examined for their efficacy against mycotoxigenic *Fusarium* species (*F. graminearum*, *F. langsethiae*, *F. verticillioides*). The objectives were to assess the inhibitory effect of these compounds on growth and mycotoxin production *in vitro*, and *in situ* in artificially inoculated wheat, oats and maize with one isolate of each respectively, at different water activity (a_w_) conditions when stored for up to 20 days at 25 °C. *In vitro*, 200 ppm of either PTS or PTSO reduced fungal growth by 50–100% and mycotoxin production by >90% depending on species, mycotoxin and a_w_ conditions on milled wheat, oats and maize respectively. PTS was generally more effective than PTSO. Deoxynivalenol (DON) and zearalenone (ZEN) were decreased by 50% with 80 ppm PTSO. One-hundred ppm of PTS reduced DON and ZEN production in wheat stored at 0.93 a_w_ for 20 days, although contamination was still above the legislative limits. Contrasting effects on T-2/HT-2 toxin contamination of oats was found depending on a_w_, with PTS stimulating production under marginal conditions (0.93 a_w_), but at 0.95 a_w_ effective control was achieved with 100 ppm. Treatment of stored maize inoculated with *F. verticilliodies* resulted in a stimulation of total fumonsins in most treatments. The potential use of such compounds for mycotoxin control in stored commodities is discussed.

## 1. Introduction

There has been interest in the use of essential oils (EOs) and extracts derived from plants to control food spoilage microorganisms, especially mycotoxigenic moulds, as an alternative to traditional preservatives based on aliphatic acids [1]. However, an important aspect to consider when utilizing natural plant extracts for control of mycotoxigenic spoilage fungi is whether they are classed as food grade. In addition, many studies have studied effects on germination and growth of spoilage mycotoxigenic fungi, while neglecting impacts on mycotoxin production, especially *in situ*. 

Onion and garlic, both members of the *Allium* family, have received attention as extracts from these two plant species have been found to have significant antimicrobial properties [1]. Their antifungal efficacy has been studied despite the relative instability of some of their compounds or their strong odour. Yin and Tsao [2] found garlic, out of seven *Allium* plants, to be the most effective against three *Aspergillus* species. Some inter-species differences in efficacy were previously noted, with higher concentrations of plant extracts required for control of *Aspergillus flavus* and *Aspergillus fumigatus* than *Aspergillus niger*. Yoshida et al. [3] suggested that the antifungal activity of garlic was due to the compounds allicin and ajoene. Benkeblia [4] observed growth inhibition of *A. niger* and *Penicillium cyclopium* by red onion and garlic EOs, while higher concentrations (200–500 mL/L) of green and yellow onion EOs were required for control the fungal pathogen *Fusarium oxysporum.* Singh and Singh [5] studied the effect of *Allium sativum* extracts and other plant extracts on the growth of *A. flavus* and aflatoxin production, but only in liquid cultures. Addition of the extract at the beginning of the incubation period showed 85% inhibition in mycelial biomass and complete inhibition of aflatoxin production. However, a later addition only gave marginal control of toxin biosynthesis. Liquid culture systems are relatively artificial as the aim should be to try and develop intervention strategies to control mycotoxigenic fungi under similar conditions found in different agrifood commodities or under simulated nutritional conditions relevant to where the target control measures are going to be instituted.

In the last decade, some compounds have been extracted from *Allium sativus* and been successfully utilised as antimicrobial preservatives in a number of products, especially in cheese production and as a fruit coating. The two extracted compounds being used are propyl propane thiosulfonate (PTS) an organosulphate, and propyl propane thiosulfinate (PTSO). Formulations of these compounds are now commercially available as Proallium, especially as a coating for extension of fruit shelf-life. While they are considered effective anti-microbials, very little detailed information is available on the efficacy of these compounds against mycotoxigenic fungi, either *in vitro* or *in situ*. 

Previous studies to compare some EOs and antioxidants to control *P. verrucosum* and ochratoxin A (OTA) production showed that of those tested only resveratrol was effective at controlling populations of the mycotoxigenic species and inhibiting OTA production in stored wheat grain under different storage conditions [6]. Environmental factors were also shown to have a significant influence on the relative control achieved. In addition, this study showed that sometimes under sub-optimal concentrations of these compounds, some stimulation of toxin production occurred despite growth being significantly reduced. This has been suggested to be due to a combination of water and physiological stress caused by the antifungal agent itself which may stimulate secondary metabolite production as a defence response [7,8,9,10].

Some very early studies suggested thiosulfinates had potential applications as anti-microbials, although very focused on their use in post-harvest grain preservation [11]. Some extracts of garlic and onion have been shown to have promising efficacy against *Aspergillus* and *Penicillium* species and in some cases mycotoxin production. However, very limited information is available on the efficacy of such EOs on *Fusarium* species. In addition, such compounds have rarely been assessed on naturally contaminated grains where a range of different species may be encountered. 

The objectives of this study were to study the effect of two garlic-derived compounds (PTS, PTSO) for the control of (a) fungal growth and (b) mycotoxin production by *Fusarium graminearum* (Deoxynivalenol, Zearalenone; wheat), *F. langsethiae* (T-2/HT-2 toxins; oats) and *F. verticilioides* (Fumonisins; maize) for the first time. Experiments were initially conducted *in vitro* for a preliminary assessment of these two compounds to determine the effective concentrations for control of growth and mycotoxin production. Subsequently, *in situ* experiments were carried out with inoculation of wheat, oats and maize with the relevant mycotoxigenic species and treatment with each of these two compounds based on *in vitro* results and stored at 25 °C under different moisture content conditions for up to 20 days to examine effects on mycotoxin production.

## 2. Results

### 2.1. In Vitro Efficacy of PTS and PTSO Garlic-Derived Compounds against Fungal Growth

**Figure 1:**
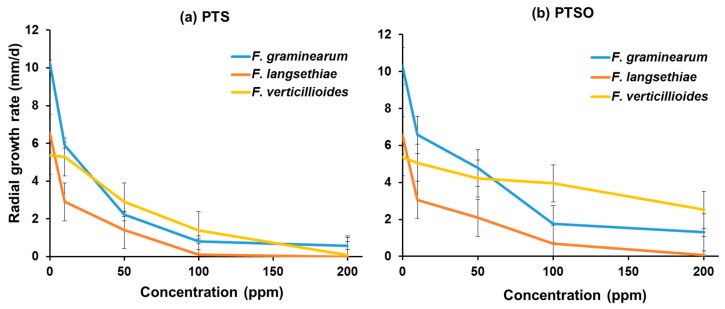
Effect of 0–200 ppm (**a**) Propyl Propane Thiosulfonate (PTS) and (**b**) Propyl Propane Thiosulphonate (PTSO) on the *in vitro* (2% wheat agar medium) on the radial growth rate (n = 6) of an isolate of *F. graminearum, F. langsethiae* and *F. verticillioides*. Vertical bars indicate the standard error of the means. For PTS effects on growth: *F. graminearum*: H (4, N = 30) = 26.612, *p* < 0.001; *F. langsethiae*: H (4, N = 30) = 27.647, *p* < 0.001; *F. verticillioides*: H (4, N = 30) = 26.522, *p* < 0.001; for fungal species: H (2, N = 90) = 3.176, *p* = 0.2004. For PTSO effects on growth: *F. graminearum*: H (4, N = 30) = 27.451, *p* < 0.001; *F. langsethiae*: H (4, N = 30) = 27.877, *p* < 0.001; fungal species: H (2, N = 90) = 12.926, *p* = 0.002.

Figure 1 shows the effect of 0–200 ppm PTS and PTSO on the *in vitro* radial growth rates of *F. graminearum, F. verticillioides* and *F. langsethiae.* Both PTS and PTSO had very good inhibitory effects on the growth of the isolate of each of these species studied and this increased with concentration. Complete inhibition of the growth of *F. langsethiae* was observed with 100 ppm PTS and of *F. verticillioides* with 200 ppm. Growth of *F. graminearum* was significantly inhibited, although complete inhibition was not achieved in the range of concentrations examined. Two hundred ppm PTSO completely inhibited the growth of *F. langsethiae* and was very effective against *F. graminearum* and *F. verticillioides.* PTS was always more effective than PTSO when applied at the same concentration against the same fungal species. Table 1 shows the concentration necessary for effective dose (ED) 50 and 90% control of growth of the three *Fusarium* species.

The statistical analyses (ANOVA) showed that the effect of PTS and PTSO concentration was highly significant on the growth of the isolate of each *Fusarium* species, with significant intra-isolate differences for growth rate in the presence of the either of these two compounds.

**Figure 2:**
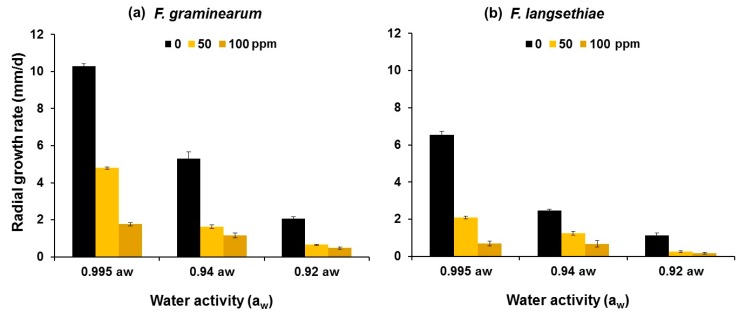
Effect of 0–100 ppm Propyl Propane Thiosulfinate (PTSO) on the radial growth rates (n = 6) of an isolate of (**a**) *F. graminearum* and (**b**) *F. langsethiae* *in vitro* (2% wheat agar medium) in relation to water activity (a_w_). Vertical bars indicate the standard error of the means. Statistical analyses for (**a**) *F. graminearum*: H (4, N = 30) = 27.451, *p* < 0.001; and for (**b**) *F. langsethiae*: H (4, N = 30) = 27.877, *p* < 0.001.

Additional studies were carried out to assess the efficacy of the two compounds at different water activity (a_w_) levels on growth of the three *Fusarium* species. Figure 2 shows an example of the effect of 0–100 ppm PTSO on the growth of *F. graminearum* and *F. langsethiae* on wheat agar media modified to three different a_w_ levels. The radial growth of both isolates of the two species was significantly inhibited as the PTSO concentration was increased and water stress was imposed (0.94, 0.92 a_w_), when compared to the unmodified control medium. The maximum percentage (%) inhibition was observed at the highest PTSO concentration and 0.995 a_w_ (~83% for *F. graminearum* and ~90% for *F. langsethiae*). ANOVA showed that the effects of either PTS or PTSO concentration, a_w_ and fungal species were highly significant on the log-transformed radial growth rate data, while the effects of the interactions between these factors were not significant. 

Table 2 summarises the effective dose (ED_50_) concentrations of PTSO only for the isolates of *F. graminearum*, *F. langsethiae* and *F. verticillioides* used in this study grown on wheat agar media modified to different a_w_ levels and 25 °C. Overall, lower concentrations of each compound were required for 50% inhibition of growth of the isolate of *F. langsethiae* than for the isolates of the other two *Fusarium* species.

### 2.2. Effects of the Compounds on In Vitro Mycotoxin Inhibition

**Figure 3:**
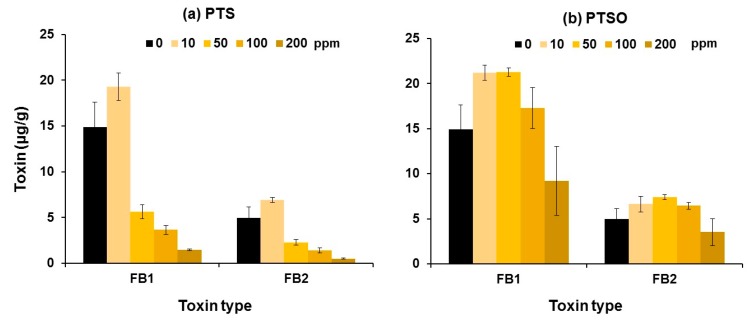
Effect of 0–200 ppm of (**a**) PTS and (**b**) PTSO on the production of fumonisins B_1_ and B_2_ by *F. verticillioides* *in vitro* at 25 °C. Vertical bars indicate the standard error of the means. For statistical analyses see Supplementary Table S2.

Figure 3 shows the effect of different concentrations of PTS and PTSO on fumonisins B_1_ and B_2_ (FB_1_ and FB_2_) production by the isolate of *F. verticillioides* on wheat-based medium at 25 °C. Two-hundred ppm of PTS inhibited FB_1_ and FB_2_ toxins by up to 90%. For PTSO, the production of both toxins was slightly stimulated with up to 100 ppm. However, the production of the two fumonisins was inhibited with 200 ppm. ANOVA showed that the effect of PTS concentration was highly significant on the log-transformed data of both FB_1_ and FB_2_. For PTSO concentration, analyses showed that there was a significant effect on FB_1_ production by the isolate of *F. verticillioides* *in vitro* but it was not significant for the production of FB_2_ (see Supplementary Table S1).

The production of deoxynivalenol (DON) by *F. graminearum* in response to exposure to either of these compounds was reduced when compared to the untreated control (Supplementary Figure S1). DON production was below the limit of detection in the 200 ppm PTS treatments. However, DON production in the 250 ppm PTSO was ~4 times more than in the control. ANOVA showed that the concentration of PTS significantly affected DON production. PTSO had no significant effect on *in vitro* DON production by *F. graminearum.*

There was a decrease in T-2 production by *F. langsethiae* as the PTS concentration was increased, and this was below the limit of detection with 200 ppm concentration (data not shown). HT-2 toxin in the same samples was not detected at ≥50 ppm PTS (Supplementary Figure S2; Supplementary Table S2). With PTSO, T-2 toxin production by *F. langsethiae* was inhibited compared to the control, although no specific pattern was observed with concentration. HT-2 toxin production was below the limit of detection in all PTSO concentrations. Statistically, the effect of PTS concentration was significant on T-2 toxin production but not for HT-2 toxin. The effect of PTSO was not significant for either of these two related type A trichothecenes. 

### 2.3. In Situ Mycotoxin Control in Stored Wheat, Oats and Maize Using PTS and PTSO 

**Figure 4:**
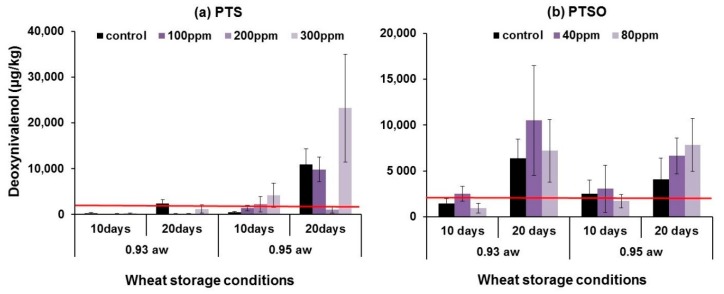
Effect of 0–300 ppm of PTS (**a**) and 0–80 ppm PTSO (**b**) on deoxynivalenol production by *F. graminearum* in artificially inoculated wheat of 0.93 and 0.95 a_w_ stored for 10 and 20 days at 25 °C (n = 2 × 3). Vertical bars indicate the standard error of the means. The red lines show the EU legislative limits (EC 1881/2006) for deoxynivalenol in unprocessed wheat for feed use (1750 µg/kg).

**Figure 5:**
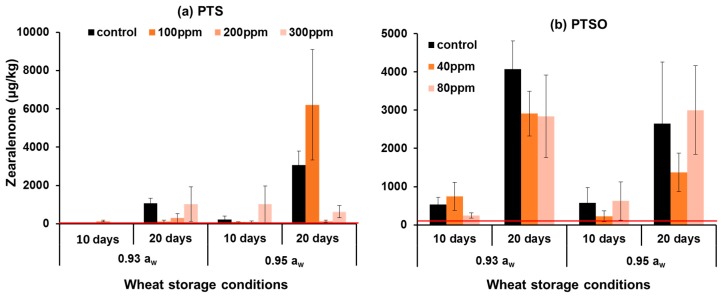
Effect of (**a**) 0–300 ppm PTS and (**b**) 0–80 ppm PTSO on zearalenone production by *F. graminearum* in artificially inoculated stored wheat at 0.93 and 0.95 a_w_ for 10 and 20 days at 25 °C (n = 2 × 3). Vertical bars indicate the standard error of the means. The red lines show the EU legislative limits (EC. 1881/2006) for zearalenone in unprocessed wheat for human use (100 µg/kg). Kruskal–Wallis ANOVA for Statistical effects for PTS concentration: H (3, N = 48) = 1.39, *p* = 0.707, grain a_w_: H (1, N = 48) = 3.44, *p* = 0.064, storage time: H (1, N = 48) = 12, *p* < 0.001; For PTSO conc.: H (2, N = 36) = 0.47, *p* = 0.789, a_w_: H (1, N = 36) = 1.48, *p* = 0.223, storage time: H (1, N = 36) = 21.34, *p* < 0.001.

Figure 4 and Figure 5 show the effects of either PTS or PTSO on DON and ZEN production in wheat modified to two different a_w_ levels, inoculated with *F. graminearum* and stored for 10 and 20 days at 25 °C. The red lines show the EU regulatory limits for each of the toxins in wheat [12] (EC 1881/2006). DON production was reduced in all the PTS-treated samples stored at 0.93 a_w_ for up to 20 days at 25 °C compared to the control. Maximum inhibition was obtained with 100 and 200 ppm PTS after 10 and 20 days storage (76% and 95%) respectively when compared with the control. In the wetter wheat samples at 0.95 a_w_ stored for 10 days DON production increased with increasing PTS concentration, while after 20 days irregular results were obtained ranging from 90% inhibition of DON production with 200 ppm PTS to >100% stimulation at 300 ppm. In the PTSO treatments, a small reduction in DON contamination (~33%) was observed with 80 ppm PTSO in wheat stored for 10 days at both a_w_ levels. With lower concentration of PTSO (40 ppm) there was a stimulation of toxin production when compared to the control. After 20 days storage stored wheat treated with PTSO had toxin levels higher than in the controls at both a_w_ levels. ANOVA showed that the effects of PTS concentration, a_w_ and storage time significantly affected log(DON) production by *F. graminearum* in the stored wheat treatments (see Supplementary Table S3). However, interactions between these factors was not significant. For PTSO, ANOVA showed that concentration and aw significantly affected log (DON) production, while the effects of storage time and interactions between factors were not significant. 

Figure 5 shows the effect of PTS and PTSO on ZEN production by *F. graminearum* in stored wheat. The most effective control of ZEN production by PTS was 100ppm at 0.93 a_w_ with 89% control after 20 days storage. In the wetter 0.95 a_w_ grain 100 ppm of PTS inhibited ZEN production by about 64% after 10 days storage. However, after 20 days storage, 200 ppm PTS was required for 96% ZEN control. Overall, the most efficient PTS concentrations for the control of both toxins (DON, ZEN) was 100 ppm PTS in the 0.93 a_w_ stored wheat, and 200 ppm for the wetter stored wheat (0.95 a_w_). PTSO at 40 and 80 ppm reduced ZEN contamination of stored wheat inoculated with *F. graminearum* at both 0.93 and 0.95 a_w_ by 30–60% after 20 days storage. Statistically, the Kruskal–Wallis analyses (non-parametric analyses) showed that there was no significant effect of PTS concentration or grain a_w_ on ZEN production, while storage time was highly significant. For PTSO, concentration or storage a_w_ were not significant for ZEN production, while storage time was highly significant. 

**Figure 6:**
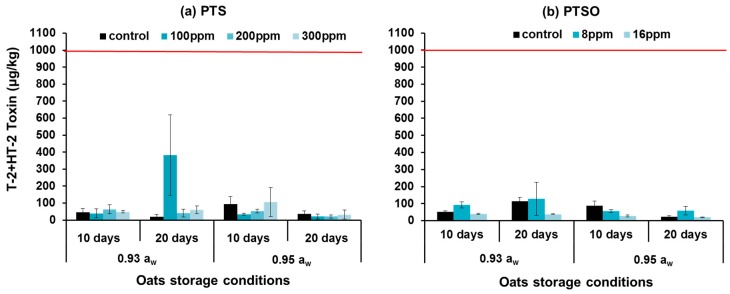
Effect of (**a**) 0–300 ppm aqueous PTS and (**b**) 0–16 ppm PTSO on T-2+HT-2 toxin production by *F. langsethiae* in artificially inoculated oats of 0.93 and 0.95 a_w_ and stored for 10 and 20 days at 25 °C. Vertical bars indicate the standard error of the means. The red lines show the EU recommended for maximum limits (EC. 165/2013) for sum T-2 and HT-2 toxin in unprocessed oat for human use (1000 µg/kg).

Figure 6 shows the T-2 + HT-2 toxins in stored oats inoculated with *F. langsethiae* and treated with 0–300 ppm PTS and 0–80 ppm PTSO for up to 20 days. The red line shows the indicative directive with regard to limits more commonly established in Europe for the sum of T-2 + HT-2 toxins [13] (EC 165/2013). In all cases, the toxin levels produced were below the indicative levels suggested by the EU. Statistical analyses showed that the effects of PTS concentration, and storage time had no significant effect on log(T-2 + HT-2) toxin contamination of stored oats by *F. langsethiae* (see Supplementary Table S3) The effect of a_w_, and interaction between a_w_ × storage time were significant. For PTSO, ANOVA showed that concentration and a_w_ had significant effects on the production of these two toxins while the effect of storage time and interactions between the factors were not significant. 

**Figure 7:**
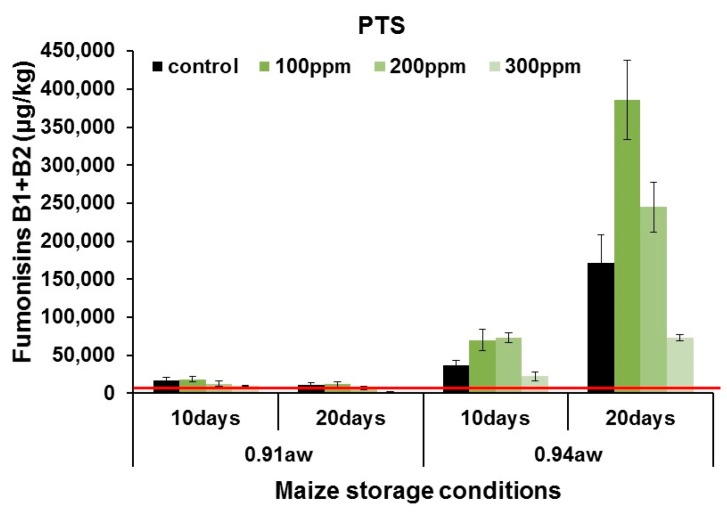
Effect of 0–300 ppm aqueous PTS on total fumonisins production by *F. verticillioides* in artificially inoculated maize stored at 0.91 and 0.94 a_w_ for 10 and 20 days at 25 °C. Vertical bars indicate the standard error of the means. The red lines show the EU legislative limits (EC. 1881/2006) for fumonisins (FB_1_ + FB_2_) in unprocessed maize for human use (2000 µg/kg).

Figure 7 shows the effect of treatments on the production of total fumonisins B_1_ + B_2_ (FUMs) in maize rewetted to 0.91 and 0.94 a_w_ and inoculated with *F. verticillioides* spores and treated with 0–300 ppm PTS, Only PTS was studied in these assays because of the limited efficacy of PTSO in controlling FUMs production *in vitro* (see Figure 3). The red line shows the EU legislative limits established for the sum of FUMs in maize. At 0.91 a_w_ PTS was effective with up to 80% control achieved after 20 days with 200–300 ppm treatment. This was also below the relevant EU limit. However, in wetter maize stored at 0.94 a_w_ most treatments were ineffective in controlling FUMs contamination. Indeed there was a stimulation in FUMs, especially with 100–200 ppm PTS, regardless of a_w_ or storage time. There was a statistically significant effect of PTS concentration, maize a_w_ and storage time on the logarithm of total FUMs production (see Supplementary Table S3). The interaction of a_w_×storage time and a_w_ × PTS concentration were also significant. However, the interactions of PTS × storage time and interaction between all three factors was not significant. For PTSO, none of the single factors or interacting factors were significant except for a_w_ × storage time was significant.

## 3. Discussion

### 3.1. In Vitro Efficacy of PTS and PTSO

This is the first study to evaluate the efficacy of the garlic-derived compounds PTS and PTSO for control of fungal growth and mycotoxin production by isolates of the three important mycotoxigenic *Fusarium* species *in vitro*. The present study has shown that solutions of either PTS or PTSO were effective at inhibiting the *in vitro* growth of each isolate of the three *Fusarium* species studied. The inhibitory effect increased with concentration and generally, the level of inhibition was species-dependent. *F. langsethiae* was the most sensitive *Fusarium* species to both compounds with complete inhibition of growth with 100 and 200 ppm of PTS and PTSO respectively. 

Our results suggest that there is differential sensitivity of *Fusarium* species to PTS and PTSO. Thus, higher ED_50_ concentrations of the two garlic-derived compounds were required for the isolate of *F. verticilloides* when compared to *F. gaminearum* and *F. langshetiae*. Previously, 200 ppm of star anise extract was needed to inhibit growth of *F. verticillioides* completely when compared to effects on *F. solani*, *F. oxysporum and F. graminearum* where only 100 ppm was required [14]. Similarly, Morcia et al. [15] observed lower ED_50_ values of seven EOs were needed in the case of *F. langsethiae* when compared to effects on *F. graminearum* and *F. sporotrichioides*. However, most of these studies did not assess interactions with water availability which is critical for determining efficacy, especially in relation to toxin control [14,15,16,17,18,19,20,21,22]. It is also difficult to compare the dose-effect relationships obtained between species across the studies because different times and doses, and different matrices were used, often nutritionally unrelated to the commodity in which *in situ* control was required. 

In addition, different EO extracts have different active ingredients and thus do not have the same efficacy against the same species. For example, the effect of EOs belonging to five botanical families (Umbeliferae, Labiateae, Compositeae, Rosaceae and Lauraceae) against *F. moniliforme* (=*F. verticillioides*) was evaluated [16]. Growth was completely inhibited with 500 ppm of anise, thyme and cinnamon, 2000 ppm of marigold, and 3000 ppm of the other extracts examined. Overall, no significant differences between these botanical families were found. Also, complete inhibition of this *Fusarium* species was achieved with 600 ppm of thyme, 800 ppm of basil and lemongrass and 1000–2500 ppm of ginger [17,18]. These differences could be due to the different tolerances between strains, but also because of the different compositions and purity of EOs obtained from the different plants. Elhouiti et al. [23] studied the chemical composition of leaves and flowers of *Rhanterium adpressum*, harvested at different times over three years. They observed that the percentage of the leading chemical groups changed according to the month of extraction. Also, the EOs produced by the flower had better inhibitory activity than the leaf extracts (MICs, 6–10 ppm vs 11–14 ppm) against *F. culmorun* and *F. graminearum* respectively. Kurita and Koike et al. [24] pointed out that the magnitude of the antifungal effect was related to the functional groups and they proposed a scale of antifungal potency of chemical groups from the best to worst being phenols > alcohols > aldehydes > ketones > ethers > hydrocarbons. However, these previous studies did not evaluate the impact that changing water availability might have on the relative control achieved.

In terms of control of mycotoxin production, both PTS and PTSO reduced DON, ZEN, T-2, HT-2 and FUMs production by the relevant *Fusarium* species when compared to the controls. There were some differences in efficacy between PTS and PTSO. The latter compound only controlled FB_1_ and FB_2_ production at 200 ppm, with >4 times the amount of PTS required, compared to PTSO to obtain the same inhibitory effects. Generally, PTS was more effective in controlling mycotoxin production than PTSO, except in the case of T-2 toxin where the latter compound was more effective at <200 ppm. Similarly, HT-2 toxin was completely inhibited at all PTSO concentrations, while for PTS ≥50 ppm was required. It was also generally observed that higher PTS concentrations (200 ppm) completely inhibited the production of all toxins, except in the case of the FUMs.

Previous studies have suggested that complete inhibition of FUMs could be achieved with 6 ppm of 3-carene, D-limonene and B-ocimene [21]. Also, ginger EOs inhibited FB_1_ (2500 ppm), FB_2_ (2000 ppm) and DON (2000 ppm) production [17,22]. Lower doses of extracts of *R. adpressum* (0.25 ppm) significantly inhibited production of type B tricothecenes (3-acetyl deoxynivalenol, 15-acetyl deoxynivalenol and fusarenon X) [23]. Morcia et al. [15] reported that the seven EOs (cuminaldehyde, cinnamaldehyde, lemon oil, citral, limonene, bergamot and citroneall) had a variable effect on the biosynthesis of T-2 and HT-2 by *F. langsethiae* and *F. sporotrichioides*. 0.1 mL of bergamoil/mL reduced T-2 and HT-2 toxin produced by the former species but stimulated production by the latter species. However, again, few of these studies included the impact of a_w_ stress on the efficacy of the EOs. 

### 3.2. In Situ Efficacy of PTS and PTSO against Mycotoxin Production in Stored Grain

The a_w_ levels chosen for the wheat, oats and maize storage studies was based on the marginal and optimum a_w_ levels for growth and mycotoxin production by the isolates of these three species from previous studies [25,26,27]. Drier conditions of <0.90 a_w_ are considered very marginal for colonisation by *Fusarium* species of wheat, oats or maize. Treatment of wheat with 100 ppm of PTS resulted in 76–94% control of DON, depending on the storage a_w_. Better results were obtained in the 0.93 a_w_ treatment where colonisation was slower than in the 0.95 a_w_ treatment. PTS was also effective in controlling ZEN production, by reducing the production to below or near the applicable EU limits after both 10 and 20 days storage respectively. However, it should be noted that under some storage conditions intermediate PTS concentrations stimulated mycotoxin production. 

Overall, PTS was not efficient in controlling the production of T-2 and HT-2 toxins in artificially inoculated oats at 0.93 a_w_, particularly in samples stored for 20 days. In contrast, at 0.95 a_w_ reasonably good control with 100 ppm PTS for T-2 and HT-2 production was achieved after 10 days storage, and with 200 ppm after 20 day storage. For control of FUMs, 300 ppm PTS was necessary in stored maize at 0.91 a_w_ and 0.94 a_w_ resulting in 39–80% inhibition when compared to the control samples. The control achieved was below the EU legislative limits but only in the 0.91 a_w_ treatments. In moist maize, much higher concentrations would be required to try and control FUMs to below the EU legislative limits, especially if destined for feed use. 

Treatment of wet grain with PTSO was generally capable of reducing mycotoxin contamination after storage at 0.93–0.95 a_w_ for 10–20 days compared to the untreated control samples. Thus, where water ingress might occur in a silo this compound would be effective at controlling toxin contamination in stored cereals produced by *Fusaria*, in the short to medium term. DON contamination in wheat treated with 80 ppm PTSO and stored for 10 days had 1/3rd less toxin than the control, which was also below the relevant EU limits. However, this treatment was not efficient for extended storage beyond 20 days with the exception of ZEN, which was more effectively reduced, even after 20 days storage. Better control was observed with 40 ppm PTSO (48–60%) in relatively moist wheat stored at 0.95 a_w_. However, all the treatments contained ZEN levels above the applicable EU limits. 

Overall, PTSO was more efficient in controlling the production of T-2/HT-2 toxins by *F. langsethiae* in oats, with as little as 16 ppm required. This treatment was generally more effective against HT-2 toxin (8–78%) than T-2 toxin (18–42%) and this may be important, as often T-2 toxin is converted rapidly to HT-2 toxin and thus control of this toxin is important. 

Previously, Soliman et al. [16] examined the efficacy of low concentrations (0.1–2.0 ppb) of the more efficient EOs tested *in vitro* (anise, cinnamon, spearmint and thyme) on FUMs contamination of stored wheat over 8 week storage periods. They claimed that low doses (0.1 ppb) completely inhibited the biosynthesis of FUMs after 2 weeks. Thyme EO was shown to have the highest anti-mycotoxigenic activity and the best control of growth. Although water availability was not considered, the concentrations appear to be very low for achieving control. In addition, *F. verticillioides* colonisation is more important in maize where it is primarily responsible for FUM contamination. Venkatesh et al. [21] suggested that use of guggul EO at 10 ppm treatment of maize at 28 °C for 10 days reduced FUMs from 42.5 to 2.6 ppm; with complete inhibition of FB_1_ achieved with 200 ppm of star anise or 50 ppb of allyl isothiocyanate [14,28]. Allyl isothiocyanate was found to inhibit the production of FB_1_ by *F. verticilloides* [2,28]. 

These cereals are naturally contaminated with a mixture of toxigenic fungi as part of the mycobiota. Thus, the differential effect on different Fusarium species would also apply to other toxigenic species such as *Penicillium verrucosum* (ochratoxin A producer) or *Aspergillus* species. Thus, consideration should be given to changes in the ratio of mycotoxins which might occur when treated with different preservatives. Recently, Giorni et al. [29] showed that in ripening maize co-inoculated with mixtures of *F. graminearum*, *F. verticillioides* and *Aspergillus flavus* influenced the relative contamination of the maize cobs with deoxynivalenol, FUMs and aflatoxins. Interactions between non-toxigenic mycobiota and has also been shown to influence the relative contamination with different mycotoxins in both wheat grain and in grape-based matrices [30].

The *in situ* storage studies have been done for a maximum of 20 days. For longer term storage periods of 6–9 months it may be necessary to use a slightly increased treatment concentration of such compounds for ensuring that control can be maintained. This would have an impact on the relative economic costs of treatment which would have to be considered in the context of the overall inputs into management of grain for food and feed use post-harvest. 

## 4. Conclusions

This was the first detailed examination of these two compounds for control of growth and mycotoxin contamination by *Fusarium* species *in vitro* and in artificially inoculated stored wheat, oats and maize under different temporal and water availability conditions. Overall, *in vitro* efficacy should include important parameters such as water availability, temperature and perhaps pH stress to identify the most effective candidates for control of colonisation, and more importantly, mycotoxin production. Potential efficacy was demonstrated and identified against isolates of three different *Fusarium* species. However, efficacy *in situ* was not as effective as that observed *in vitro*. In addition, the efficacy of the treatments depended on the specific “*Fusarium* species-toxin” pathosystem. Differences were observed with regard to the production of different mycotoxins by an isolate of a single fungal species when inoculated into naturally contaminated cereals. However, the right concentrations need to be used for effective control to be achieved. This depended on the water availability and the mycotoxigenic species involved. 

Both garlic-derived compounds tested in this study (PTS and PTO) are liquids and water soluble and can thus be applied to grain prior to storage for post-harvest control of spoilage mycotoxigenic fungi. Garlic extracts have been approved for use as a pesticide (although currently under re-evaluation) and commercial products are available based on such extracts. Certainly, the use of odourless versions of these compounds could be effectively used for food applications. In addition, the existing compounds could also be used for animal feed applications where often moist grain needs to be preserved for the short to medium term prior to use. 

## 5. Materials and Methods

### 5.1. Preparation of Stock Solutions of Chemical Compounds

Stock solutions of the following compounds were prepared in sterile distilled water. 

(a) Propyl propane thiosulfonate (PTS): This organosulfonate compound is obtained from the decomposition of initial compounds present in garlic bulbs (*Allium sativum*) and was kindly provided by DOMCA SA, Granada, Spain. A stock solution of 5000 ppm was prepared by dissolving 1.1 g PTS (90% PTS, Domca, S.A., Granada, Spain) (Mousala SL., 2006) into a 200 mL container containing 200 mL of sterile distilled water and vigorously shaking. Due to the oily nature of PTS the stock solution had the appearance of a stable water emulsion. A second stock solution of 20000 ppm was prepared by dissolving 2.2 g PTS into 100 mL of a mixture of ethanol:H_2_O (80:20). This solution was clear.

(b) Propyl propane thiosulfinate (PTSO): A stock solution of 10000 ppm was prepared by dissolving 1.1 g PTSO (90% PTSO, Domca, S.A., Granada, Spain) (Mousala SL., 2006) into a 100 mL container containing 100 mL sterile distilled water and vigorously shaking. Due to the oily nature of PTSO the stock solution had the appearance of a water emulsion. A second stock solution of 20,000 ppm was prepared as for PTS.

### 5.2. In Vitro Studies: Fungal Species, Media, Inoculation and Measurements of Growth and Mycotoxin Production

*Fusarium graminearum* isolate L1-2/2D (wheat; DON, ZEN), *F. langsethiae* strain 2004/59 (oats; T-2 + HT-2) and *F. verticillioides* isolate MPVP 294 (maize; FUMS) were used in this study. The strains were all maintained on Malt Extract Agar (MEA) media (OXOID, malt extract, 30; mycological peptone, 5; agar, 15 g/L). These isolates were kindly supplied by Prof. S. Edwards, Harper Adams University and Prof. P. Battilani, Catholic University of Italy, Piacenza, Italy). They have all been examined previously for mycotoxin production and shown to be high producers of the respective mycotoxins in previous studies [31,32].

For the *in vitro* trials a 2% milled wheat medium was prepared by adding 2% milled wheat and 2% agar (OXOID Ltd, Basingstoke, England) to water to obtain the basal medium. For the initial screening, concentrations of PTS and PTSO in the range 10–200 ppm were used by adding the necessary stock solutions to the molten cooled medium, shaking vigorously and then pouring the media into 9 cm Petri plates (15 mL per plate). The basal 2% media had a water activity (a_w_) value of 0.995. This basal medium was modified by replacing water with different amounts of mixtures of glycerol/water solution to the milled cereal + agar to obtain the target a_w_ values of 0.92, 0.94 and 0.98 (20.7, 15.4 and 4.9 g glycerol/50 mL of water, respectively) without diluting the nutritional status of the media. These a_w_ levels represent the range over which these *Fusaria* can effectively grow [33]. The media were all checked with an Aqualab TE4 to confirm the actual a_w_ levels were achieved.

Agar plugs (4 mm diameter) cut from the margin of 10-day-old cultures with a sterile cork borer, were used as an inoculum for the *in vitro* trials. Three replicate per treatment and replicate plates were centrally inoculated with the inoculum agar plugs. All experiments were repeated once. Each a_w_ treatment and replicates were stored in separate polyethylene bags to maintain the environmental conditions over the experimental period. 

The treatments and replicates were all incubated at 25 °C for 10 days, or until the Petri dishes were completely colonised by the fungi. Each concentration of the PTS and PTSO treatments were kept separately in polyethylene bags to avoid cross-contamination. Two diameters of the colonies formed (at right angles of each other) were measured daily and compared against the diameters of the controls. From these data the relative growth rates were calculated and the effect of different concentrations was calculated. The percentage inhibition of mycelial growth of the *Fusarium* species was determined at each different chemical compound concentration and the different water activities. 

For mycotoxin analyses on the tenth day of incubation, agar plugs (5, 5-mm diameter) were cut out from each of the replicate plates diagonally across the colony. The agar plugs were placed in 2-mL safe-lock Eppendorf® tubes (Eppendorf AG, Hamburg, Germany), their weight was recorded and frozen at −40 °C for subsequent toxin analysis. The extraction and analysis of the relevant toxins for each fungal species were performed according to the methods described later.

### 5.3. In Situ Studies with Stored Cereal Grain

*Fungal inoculum:* Cultures of the above fungal species were prepared on MEA and incubated at 25 °C for 10 days. A Tween 80 solution was prepared by addition of one drop of Tween 80 (ACROS organics) in 100 mL sterile water. Spore suspensions were prepared by gently scraping the culture surface with a sterile spatula and transferring the spores into sterile 25 mL Universal glass vials containing the water + Tween 80 solution. The spore suspensions were filtered through glass wool in order to remove any mycelial fragments. The spore concentration was determined using a haemocytometer (Olympus BX40 microscope, Microoptical Co.; slide Marienfeld superior, Germany; microscope glass cover slips, No 3, 18 × 18mm, Chance Proper LTD, Smethwick, UK) and adjusted by dilution to 10^7^ spores/mL. Naturally contaminated wheat, oats and maize were artificially inoculated with these suspensions for the *in situ* storage experiments.

*Grain equilibration:* Water adsorption curves were prepared for each grain type. The amount of water required to accurately modify these cereals to 0.91, 0.94 (maize) and 0.93 and 0.95 a_w_ (wheat, oats) was determined from these curves. Initially, each grain type (approx. 1 kg) was taken from a 25-kg bag and placed in a Duran bottle (2.5 L). The initial moisture content was known from the moisture adsorption curves. The grain was randomly divided into batches of 100 g in Duran flasks (1 L). These were labeled for each treatment condition and the required amounts of sterile water added to each one including the treatment PTS or PTSO stock solution, shaken vigorously and sealed. They were placed at 4°C to equilibrate overnight. The treatments were then inoculated with 1-mL spore suspension containing ~10^5^ spores/mL of the individual *Fusarium* species and thoroughly mixed using a roller mixer in order for the spores to become dispersed throughout the grain mass. For each grain type, 15 g was weighed into surface sterilized 40 mL vials (Chromacol Ltd., London, UK) with microporous lids to obtain six replicates per a_w_ treatment and stored in sandwich boxes for up to 20 days. The equilibrium relative humidity (ERH) was maintained by including 2 × 500 mL of a glycerol-water solution in beakers to maintain the treatment a_w_ levels. The experiments were carried out twice with six replicates per treatment. 

For each experiment, after 10 and 20 days storage, three replicates were destructively removed from storage chambers and frozen at −20 °C for subsequent toxin analysis. Grain samples were oven-dried for 24–48 h at 60 °C, milled and then extracted and analysed as described later. 

### 5.4. Mycotoxin Analyses

#### 5.4.1. Equipment Description

High performance liquid chromatography (HPLC) were used to quantify DON, T-2 and HT-2 from media. In addition, liquid chromatography tandem mass spectrometry (LC-MS/MS) were used to quantify FUMS from media and *Fusarium* toxins from grains. HPLC used consisted of an Agilent 1200 Series system equipped with a UV diode array detector (DAD) set at 220.4 nm (Agilent Technologies, Palo Alto, CA, USA). The column used for the chromatographic separation was a Phenomenex^®^ Gemini C_18_, 150 mm × 4.6 mm, 3 µm (Phenomenex, Macclesfield, UK) preceded by a Phenomenex^®^ Gemini 3 mm guard cartridge and the column temperature was set at 25 °C. LC-MS/MS used consisted of an QTrap 5500 LC-MS/MS System (Applied Biosystems, Foster City, CA, USA) equipped with a TurboIonSpray electrospray ionization (ESI) source and an 1290 Series HPLC System (Agilent, Waldbronn, Germany). Chromatographic separation was performed at 25 °C on a Gemini^®^ C_18_-column, 150 × 4.6 mm i.d., 5 µm particle size, equipped with a C_18_ 4 × 3 mm i.d. security guard cartridge (all from Phenomenex, Torrance, CA, USA).

#### 5.4.2. *In Vitro* Extraction and Analysis

Agar plugs were removed from media and placed in the 2-mL safe-lock Eppendorf® tubes. After that, the weight of the agar plugs were register.

##### Deoxynivalenol

The extraction was performed using 1-mL acetonitrile:water (AcN:H_2_O) (84:16), the mixture more commonly used for trichothecenes extraction [34] and the tubes were shaken in an orbital shaker at 200 rpm in the dark for 60 min at 25 °C. The extract was transferred to a new tube and oven-dried overnight at 60 °C. Subsequently, it was redissolved in 1 mL 90:10 (H_2_O: AcN) and vortexed for a few seconds. The cleaning step involved the addition of 150 mg/mL Alumina directly into the redissolved extract followed by vortexing the mixture for 15 s. The treated extract was then filtered through a 0.22 µm Millipore filter (Minisart, Sartorius, Germany) into an amber silanised LC vial and inserted into the LC-DAD for analysis. The chromatographic analysis was performed in the gradient mode, using water (solvent A) and acetonitrile (solvent B). The starting composition of the mobile phase was 5% B, at a flow rate of 0.5 mL/min held for 2 min. The composition was then gradually changed to 25% B over 15 min and maintained for further 3 min. Then it increased gradually to 30% B over 3 min at the same flow rate. The composition was then changed to 99% B during 1 min with a flow rate of 1 mL/min in this case, in order to achieve a fast cleaning step and maintained at 99% B for 4 more minutes. Afterwards the composition of the mobile phase was changed linearly to 5% B in 1 min at a flow rate of 1 mL/min and held for 4 min for further cleaning. In the last step, the composition was maintained at 5% B, but the flow rate changed to 0.5 mL/min for 1 min, in order to be the same as the starting composition of the mobile phase for the following chromatographic run. The injection volume was 50 μL. The total time for the analysis of each sample was 35 min. DON was eluted from the column at 16.2 min. The LOD was 4 µg/kg. The mean recovery for DON using this method was 63.2 ± 2.8%. 

##### T-2 and HT-2

Extraction method used was Medina et al. [32] with modifications. The extraction was performed using 1 mL acetonitrile:water (AcN:H_2_O) (84:16) and the tubes were shaken for 1 h at 150 rpm at 25 °C in the dark in an orbital shaker. The samples were then centrifuged at 1150× *g* for 15 min. The extract was filtered through a 0.2 µm Millipore filter (Minisart, Sartorius) directly into an HPLC silanised amber vial and injected in the chromatograph. The analysis was performed in the gradient mode with a mobile phase of AcN:H_2_O at a flow rate of 1 mL/min and the conditions were 3 min 30% AcN, changed linearly to 55% AcN over 18 min, changed to 99% AcN in 1 min and held to 99% AcN for 5 min. The LOD was 4 and 5 µg/kg for T-2 and HT-2. The mean recoveries for this method were 99 ± 1.53% for T-2 toxin and 101.28 ± 3.11% for HT-2 toxin. 

##### Fumonisins

The extraction was performed using 1 mL of extraction solvent AcN:H_2_O:Acetic acid (79:20:1) and the tubes were shaking 250 rpm in the dark for 1 h. The extracts were filtered through a 0.22 µm Millipore filter (Minisart, Sartorius) into new tubes, and dried in an oven at 60 °C for 24 h. The dried extracts were redissolved in a mixture of AcN:H_2_O (1:1) containing 1% acetic acid. The individual fumonisins were quantified using LC-MS/MS according to the method of Vishwanath et al. [35]. LOD was 25 µg/kg with a recovery rate of 57% (FB_1_) and 70% (FB_2_). 

#### 5.4.3. *In Situ* Extraction and Analysis

The initial mycotoxin contamination levels were quantified and this was taken into account when calculating the results of the treatments and appropriately corrected. The mean contamination levels were: DON, 0.233 µg/kg; T-2 toxin, 9.07 µg/kg (no HT-2 toxin present); ZEN, 8.42 µg/kg, and FB_1_ 0.14 µg/kg. The grain samples were oven-dried at 60 °C for 24–48 h and then milled in a small laboratory blender (Waring Commercial, Christison, UK). Samples were analysed for *Fusarium* toxins by LC-MS/MS at the Centre for Analytical Chemistry, Department of Agrobiotechnology (IFA-Tulln, Tulln, Austria), University of Natural Resources and Life Sciences, Vienna, Austria. The analysis was performed according to the methods described by Sulyok et al. and Vishwanath et al. [35,36]. The accuracy of the method was externally checked by participation in proficiency testing organised by the Bureau Inter Professionel d’Etudes Analytiques (BIPEA; Gennevilliers, France). Z-scores were 0.4 and 0.62 for DON in two wheat samples, −0.8 and −1.09 for ZON in two wheat samples and 1.36 and 1.55 for FB_1_ and FB_2_, respectively in a sample of maize.

### 5.5. Statistical Analysis

All experiments have been performed in triplicate and repeated once. Data were analysed with Microsoft Office Excel 2007 and with the package STATISTICA 9 (StatSoft^®^, Inc. 2010. STATISTICA (data analysis software system), version 9.1. www.statsoft.com, (Tulsa, OK, USA). The standard error of the mean was calculated in all trials and it is denoted with vertical bars in the figures. 

Datasets were tested for normality and homoscedasticity using the Shapiro–Wilk and Levene test, respectively. When data failed the normality test, variable transformation was performed to try to improve normality or homogenise the variances. If still not normally distributed, it was analysed using the Kruskal–Wallis test by ranks. 

## Figures and Tables

**Table 1:** Effective dose of PTS and PTSO for 50% and 90% control (ED_50_; ED_90_ values) of growth, when compared to the untreated control, for inhibition of *F. graminearum*, *F. langsethaie* and *F. verticillioides* on 2% milled wheat agar medium at 25 °C.

Treatment (ppm)	PTS		PTSO	
Species	ED_50_	ED_90_	ED_50_	ED_90_
*F. graminearum*	33	144	52	186
*F. langsethiae*	12	80	21	113
*F. verticilloides_0_*	64	172	>200	>200

**Table 2:** Effective dose ED_50_ values (ppm) of PTSO for 50% inhibition of the growth of *F. graminearum, F. verticillioides* and *F. langsethiae* on wheat agar media of different water activities (a_w_) at 25 °C.

Treatments	*F. graminearum*	*F. verticillioides*	*F. langsethiae*
**PTSO (0.98 a_w_)**	42	189	9.5
**PTSO (0.94 a_w_)**	36	>100	50
**PTSO (0.92 a_w_)**	37	>100	32

## Supplementary Materials

The following are available online at https://www.mdpi.com/2072-6651/11/9/495/s1, Figure S1: Effect of 0–250 ppm PTSO and PTS on *in vitro* DON production by *F. graminearum* in wheat agar media at 25 °C, Figure S2: Effect of 0–250 ppm (**a**) PTSO and (**b**) PTS on the production of T-2 and HT-2 toxins by *F. langsethiae* *in vitro* at 25 °C, Table S1: One-way ANOVA for the effect of different PTS and PTSO concentrations on *in vitro* fumonisins (B_1_ and B_2_) production by *F. verticillioides*, Table S2: Kruskal-Wallis ANOVA by ranks for the effect of PTSO concentration and substrate water activity on the production of T-2 and HT-2 toxins by *F. langsethiae*, Table S3: Summary of statistical *P*-values of effects of (**a**) PTS and (**b**) PTSO on mycotoxin contamination of stored cereals.
