# Supplementary material for: Influence of Two Garlic-Derived Compounds, Propyl Propane Thiosulfonate (PTS) and Propyl Propane Thiosulfinate (PTSO), on Growth and Mycotoxin Production by Fusarium Species In Vitro and in Stored Cereals
Source: Toxins (Basel). 2019 Aug 27;11(9):495. doi: 10.3390/toxins11090495 (PMC6783911; doi:10.3390/toxins11090495)
Supplement: Supplementary file 1 [file toxins-11-00495-s001.pdf]

# Supplementary Materials: Influence of Two Garlic-Derived Compounds, Propyl Propane Thiosulfonate (PTS) and Propyl Propane Thiosulfinate (PTSO), on Growth and Mycotoxin Production by *Fusarium* Species in Vitro and in Stored Cereals

**Kalliopi Mylona, Esther Garcia-Cela, Michael Sulyok, Angel Medina and Naresh Magan ***

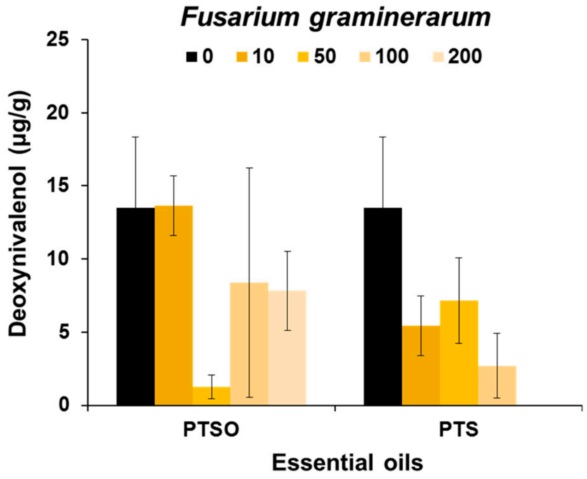


**Figure S1.** Effect of 0–250 ppm PTSO and PTS on *in vitro* DON production by *F. graminearum* in wheat agar media at 25°C. Vertical bars indicate the standard error of the means.

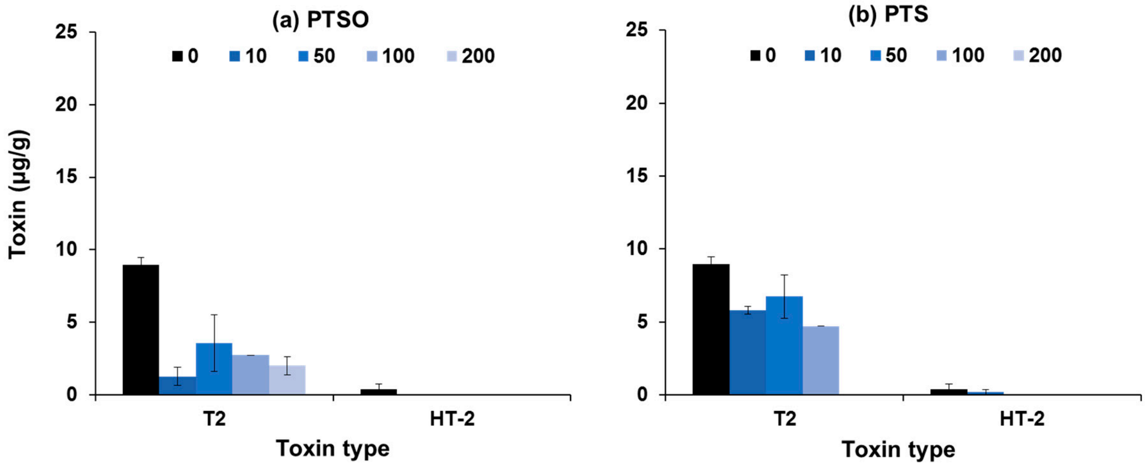


**Figure S2.** Effect of 0-250 ppm (**a**) PTSO and (**b**) PTS on the production of T-2 and HT-2 toxins by *F. langsethiae in vitro* at 25 °C. Vertical bars indicate the standard error of the means.

**Table S1.** One-way ANOVA for the effect of different PTS and PTSO concentrations on *in vitro* fumonisins ($B_1$ and $B_2$) production by *F. verticillioides.*

| | | Effect | SS | DF | MS | F | p |
|---|---|---|---|---|---|---|---|
| PTS | $logFB_1$ | Intercept | 9.169 | 1 | 9.169 | 933.238 | <0.001 |
| | | ppm | 2.450 | 4 | 0.613 | 62.346 | <0.001 |
| | | Error | 0.098 | 10 | 0.01 | | |
| | $logFB_2$ | Intercept | 1.737 | 1 | 1.737 | 109.022 | <0.001 |
| | | ppm | 2.365 | 4 | 0.591 | 37.106 | <0.001 |
| | | Error | 0.159 | 10 | 0.016 | | |
| PTSO | $FB_1$ | Intercept | 4216.142 | 1 | 4216.142 | 204.658 | <0.001 |
| | | ppm | 302.680 | 4 | 75.670 | 3.673 | 0.043 |
| | | Error | 206.009 | 10 | 20.601 | | |
| | $FB_2$ | Intercept | 502.71 | 1 | 502.71 | 154.425 | <0.001 |
| | | ppm | 28.237 | 4 | 7.059 | 2.169 | 0.146 |
| | | Error | 32.554 | 10 | 3.255 | | |

**Table S2.** Kruskal-Wallis ANOVA by ranks for the effect of PTSO concentration and substrate water activity on the production of T-2 and HT-2 toxins by *F. langsethiae.*

| | Effect | Code | Valid N | Sum of Ranks | Mean Rank |
|---|---|---|---|---|---|
| T-2 toxin | ppm PTSO | 1 | 6 | 81.000 | 13.500 |
| | | 2 | 6 | 43.000 | 7.167 |
| | | 3 | 6 | 47.000 | 7.833 |
| | $a_w$ | 1 | 9 | 77.000 | 8.556 |
| | | 2 | 9 | 94.000 | 10.444 |
| HT-2 toxin | ppm PTSO | 1 | 6 | 69.000 | 11.500 |
| | | 2 | 6 | 45.000 | 7.500 |
| | | 3 | 6 | 57.000 | 9.500 |
| | $a_w$ | 1 | 9 | 75.000 | 8.333 |
| | | 2 | 9 | 96.000 | 10.667 |

* T-2: $a_w$: H (1, N = 18) = 0.57, $p$ = 0.452; ppm PTSO: H (2, N = 18) = 5.12, $p$ = 0.077; HT-2: $a_w$: H (1, N = 18) = 0.88, $p$ = 0.349; ppm PTSO: H (2, N = 18) = 1.72, $p$ = 0.423.

**Table S3.** Summary of statistical *P*-values of effects of (**a**) PTS and (**b**) PTSO on mycotoxin contamination of stored cereals.

| | **Mycotoxin** | **logDON (wheat)** | **logT2 + HT2 (oats)** | **logFUMs (maize)** |
|---|---|---|---|---|
| **a) PTS** | Intercept | < 0.001 | < 0.001 | < 0.001 |
| | PTS ppm | 0.044 | 0.924 | 0.011 |
| | $a_w$ | <0.001 | 0.167 | 0.011 |
| | Storage time | 0.011 | 0.161 | 0.011 |
| | ppm x $a_w$ | 0.205 | 0.234 | 0.011 |
| | ppm x storage time | 0.107 | 0.241 | 0.555 |
| | $a_w$ x storage time | 0.963 | 0.043 | <0.001 |
| | ppm x $a_w$ x storage time | 0.779 | 0.380 | 0.428 |
| **b) PTSO** | Mycotoxin | DON (wheat) | logT2 + HT2 (oats) | |
| | Intercept | <0.001 | < 0.001 | |
| | PTS ppm | 0.709 | 0.070 | |
| | $a_w$ | 0.912 | 0.010 | |
| | Storage time | <0.001 | 0.296 | |
| | ppm x $a_w$ | 0.716 | 0.901 | |
| | ppm x storage time | 0.590 | 0.989 | |
| | $a_w$ x storage time | 0.615 | 0.064 | |
| | ppm x $a_w$ x storage time | 0.667 | 0.065 | |
